# Abscence of the Uterus, with a Previous History of Chronic Inversion of This Organ, Which Was Mistaken for Polypus, and Removed by Ligature

**Published:** 1877-02

**Authors:** W. R. Whitehead

**Affiliations:** Denver, Colorado


Absence of the Uterus, with a Previous History of Chronic Inversion of this Organ, which was mistaken for Polypus, and Removed by Ligature.—*By W. R. Whitehead, M.D., of Denver, Colorado.*—The removal of the uterus is of sufficient interest, under any circumstances, to merit grave consideration; but when the result of an error of diagnosis, every physician will be painfully impressed with the necessity of a careful and accura'e physical examination, before attempting to perform on the uterine organs, operations of such capital importance. This duty is also made imperative, from the fact that some eminent and usually judicious surgeons have related that this accident has occurred in their practice. I believe, therefore, that the following case will be thought worth recording:

Mrs. Hattie S., æt. 28, living a few miles from Denver, accompanied by her husband, consulted me at my office, on the 25th of last July. Mr. S. remarked that his wife had long been a sufferer, had undergone much treatment for disease of the womb, had been subjected to two or three operations on this part, and that unless I thought I could cure her, he did not wish me to undertake her case. I assured Mr. S. that I endeavored to practise my profession conscientiously, and was indisposed to make hasty promises of cure, especially in uterine diseases. This led to a few questions, and a digital and bimanual examination of the pelvic organs, and a recognition of her condition, which was further explained by a more detailed account of her previous history.

Mrs. S. menstruated at twelve years of age, and at thirteen was married. Her husband was killed a week after her marriage. Five and a half years afterward she was married to her present husband. About six months after her second marriage, and before coming to Colorado to live, she had a miscarriage at the fourth month; and she got up within three days after her sickness. It was not until a month afterward that she noticed blood to flow freely from her, and then she said, "it flowed all the time, and very badly, and continued so more or less for four years." She came under the observation of a number of Denver physicians, one of whom was Dr. R. G. Buckingham, an eminent and much respected physician, and our present mayor. In 1868, Dr. F. J. Bancroft, a prominent physician of Denver, and Dr. A. L. Justice, formerly a practitioner of this city, attended her. Dr. Bancroft informed me to-day that he and Dr. Justice diagnosed an intra-uterine fibroid tumor, having a large pedicle, which was attached to the fundus; and that they constricted the pedicle with silver wire by means of a double canula. He also remarked that the fibroid sloughed and came away in small pieces a few days afterward; but that a meddlesome nurse repeatedly pulled at the ligature, and caused inversion of the uterus. He further stated that very soon afterward another physician was called to attend the case. The patient stated that this physician, and to whom Dr. Bancroft alludes, placed a loop or ligature of wire or thread around the tumor and strangulated it, and that something came away. Finally, about the last of August, 1873, another practitioner of this city, with an assistant, used ligatures and instruments, and removed from her what seemed to be a tumor. *From that time she ceased entirely to menstruate;* but has had more or less leucorrhœa, and been under treatment for supposed uterine disease up to the time I saw her.

A digital examination revealed that the os was slightly patulous, and the lips of the cervix enlarged and indurated. Nothing by a mere digital examination led me to suspect a very unusual condition of the parts. However, by the bimanual exploration, I could not detect the body of the uterus in its normal position, or in a position of flexion or version. I passed a catheter into the bladder, and the index finger into the rectum, and found the body of the uterus to be absent; introduced the speculum and found the os to be rather everted; introduced into the cervical canal the uterine probe, which could be made to enter only three-quarters of an inch; removed the speculum and again introduced the probe into the cervical canal, and the index finger into the rectum, and approaching the end of the probe and the index finger, found in place of the uterus an indurated mass of only slight thickness interposed between the probe and finger. Both she and her husband seemed much astonished when, after my examination, I remarked that she had no womb, and that the supposed tumour which was taken from her was the uterus. I told Mr. S. that I could do nothing more for his wife than to relieve her of the leucorrhœal discharge, and for which I prescribed some powders of alum and sulphate of zinc to be used in vaginal injections.

There were some points of rare interest observed in this case, one of which was the freedom from severe bleeding until a month after the miscarriage, and which was suggestive of the production of inversion a considerable time subsequent to the premature labor. Leblanc, referred to by Thomas, and mentioned by Courty, cites a case of inversion reduced with facility soon after labor, and which was reproduced ten days afterward, and restored with difficulty. It seems to me, as Thomas observes, quite impossible to admit the occurrence of inversion of an undilated uterus; and that pregnancy, or hydrometa, or retained menses, or an intra-uterine fibroid, or some other similar dilating cause, must previously exist. Thus is explained the occurrence of inversion at variable intervals of time after pregnancy, or in women who have never been pregnant. When inversion is due to a fibroid tumor, as Courty very properly observes, such a tumor, or a polypus, with or without pedicle, is the more readily mistaken for inversion, as each is capable of causing it, and thus complicate the inversion; but it is well known that the diagnostic signs of a fibroid or polypus are usually sufficiently manifest if the proper exploration is made. By the aid of sponge-tents the interior of the uterus can be explored for small intra-uterine fibroids. With a No. 6 gum-elastic catheter, with a wire in it, and used in the manner Sims has pointed out, the uterus can be effectively explored for the largest fibroids. If the tumor be a polypus, and occupy the vagina, by the aid of the common uterine probe, and conjoined manual and digital examination, fibroids or polypi may nearly always be distinguished from inversion.

On practising the bimanual exploration on Mrs. S., had I possessed no knowledge of her antecedent history, and carelessly, as has often been done, confined my examination to a mere digital exploration, her condition would have remained undiscovered. There was, however, no peculiar merit in resorting to the bimanual examination; it is an omission not to do it. Had this examination been ever so carefully made, and without a knowledge of her previous history, I should have been liable to mistake her case for congenital absence of the uterus. Five or six years ago I was invited, with the late Prof. J. C. Nott, by Prof. Isaac E. Taylor, of New York, to examine at his office a fine-looking young woman, with every external appearance of splendid physical development, who had congenital absence of the uterus, and who presented very much the same degree of rudimental development of the cervix that was found in the case of Mrs. S. after the loss of her uterus, but without, however, the patulous os and indurated cervix.

Prof. Willard Parker's case of inversion mentioned by Thomas, which occurred during a violent effort in rolling ten-pins, and which was mistaken for polypus, and which, after removal, proved to be the uterus, with its tubes and ligaments, was not only an instructive warning against possible errors of this kind, but also shows the suddenness with which inversion may occur even years after pregnancy. Not less instructive was the case of Prof. Budd, of New York, an eminent and skilful gynæcologist, also mentioned by Thomas, and who was fully alert to the importance of not mistaking a partial inversion for polypus, yet by error removed one horn of the uterus with a part of the corresponding fallopian tube and ligament, for a supposed intra-uterine polypus.

Formerly, removal of the uterus was much practised for inversion of this organ, but in this country at least the intentional extirpation or removal of the uterus for inversion has become quite exceptional, and been replaced by more conservative methods. The procedures of Tyler Smith, of White of Buffalo, of Noeggerath, and especially of Thomas of New York, are well known. Marion Sims, a decade ago, in his work on Uterine Surgery, p. 134, remarked, in alluding to the success attending his own efforts and those of others, at restoration of the uterus, that he would hesitate a long time before removing another inverted uterus. So that the merit of success in a case of this kind, which is procured at the price of depriving a young woman of one of the most important organs of reproduction, would not justify a deliberate removal of the uterus for inversion, without repeated and intelligent trials of the known methods of reduction. Courty's method is well worth essaying, but is not so extensively known as that of White or Thomas, but is briefly described by the writer in a review of Courty's work, and is to be found in the number of the *American Journal* for January, 1872, p. 157.

An interesting question may arise regarding one of the modes of the mechanism of inversion; and which is, How far will pressure on the fundus during labor, to facilitate expulsion of the child, aid in causing inversion? I have always regarded this pressure, judiciously practised, as an invaluable aid during manual or instrumental delivery; and Barnes and others make favorable mention of it. Recently I had a case of partial inversion immediately following the expulsion of the child; the extreme shortness of the cord, together with pressure on the fundus during the last and rather ineffectual contractions of the uterus, were the cause. The uterus was immediately and readily reduced; but there was a tendency to recurrence, and I thought it necessary to give a large dose of ergot, and place the patient for an hour in the postural position on her elbows and knees, before I felt satisfied that the inversion would not be reproduced. To this, it is hardly necessary to add, that proper watchfulness was exercised during the convalescence.—*Am. Jour. Med. Sciences.*

